# Financial scarcity and financial avoidance: an eye-tracking and behavioral experiment

**DOI:** 10.1007/s00426-024-02019-7

**Published:** 2024-08-19

**Authors:** Leon P. Hilbert, Marret K. Noordewier, Lisa Seck, Wilco W. van Dijk

**Affiliations:** 1 Department of Psychology, University of Amsterdam, Amsterdam, The Netherlands; 2 Knowledge Centre Psychology and Economic Behaviour, Leiden, The Netherlands; 3 Department of Social, Economic and Organisational Psychology, Leiden University, Leiden, The Netherlands; 4 Department of Human Resource Management, Universität Duisburg-Essen Mercator School of Management, Duisburg, Germany

## Abstract

When having less money than needed, people experience financial scarcity. Here, we conducted a laboratory experiment to investigate whether financial scarcity increases financial avoidance – the tendency to avoid dealing with ones finances. Participants completed an incentivized task where they managed the finances of a household by earning income and paying expenses across multiple rounds. We manipulated participants’ financial situation such that they either had sufficient (financial abundance) or insufficient (financial scarcity) financial resources. At the end of each round, participants received an additional expense in the form of a letter. To measure financial avoidance in the form of attentional disengagement, we used an eye-tracker and assessed whether participants in the financial scarcity condition avoided looking at the expense letters. As a behavioral measure of financial avoidance, participants had the option to delay the payment of these expenses until the end of the experiment at no additional cost. Results showed no effect of financial scarcity on the eye-tracking measure, but there was an effect on the behavioral measure: Participants that experienced financial scarcity were more likely to delay payments. The behavioral finding corroborates the notion that financial scarcity can lead to financial avoidance. We explore potential reasons for the null-effect on the eye-tracking measure and discuss how future research can build upon our findings.

Trying to pay one’s expenses when having little money can lead to a feeling of financial scarcity, which is the subjective experience that financial resources are insufficient to meet demands (Shah et al., 2012; De Bruijn & Antonides, 2022). This experience can result in stress if the financial situation is seen as a threat that cannot adequately be dealt with (Van Dijk et al., 2022). In the present study, we investigated experimentally whether financial scarcity increases financial avoidance. Here, financial avoidance is the tendency to avoid dealing with one’s finances (see Hilbert et al., 2022a) and can take different forms, such as avoiding financial information (Gigerenzer & Garcia-Retamero, 2017; Golman et al., 2017; Hertwig & Engel, 2016) or delaying financial decisions (Anderson, 2003). This research extends previous correlational findings showing that financial scarcity is associated with increased financial avoidance over time (Hilbert et al., 2022a), that low economic status is associated with a general avoidance motivation (Gilbert et al., 2022), and that people tend to avoid to learn financial information if they expect it to be negative (Karlsson et al., 2009).

## Financial scarcity and financial avoidance

There are several reasons as to why financial scarcity may lead to financial avoidance. In general, negative financial information is more likely to be avoided (Karlsson et al., 2009). Compared to those who are financially well off, people facing financial problems should expect new financial information to more likely be negative. Moreover, when people experience financial scarcity, financial information might function as a scarcity cue and trigger negative emotions such as worry and shame (De Bruijn & Antonides, 2020; Plantinga, 2019). Research suggests that information eliciting such negative emotions may be avoided (Sweeny et al., 2010; see also Elliot, 2006). People also tend to neglect information if it has the potential to threaten a positive self-image and identity beliefs (Barrafrem et al., 2024), which is more likely to be the case for people that experience financial scarcity (Shah et al., 2018). In addition, a core aspect of financial scarcity is the perception of having little control over one’s financial situation (Hilbert et al., 2024; Jachimowicz et al., 2022; Van Dijk et al., 2022). People thus feel that their actions might not consistently lead to desired outcomes (Landau et al., 2015). For example, when receiving letters that are likely to contain bills, people might not open them because they feel that they do not have the financial resources to pay them. Thus, an important reason why financial scarcity might lead to financial avoidance is that people feel that learning the information might not help them resolving their problem (Howell et al., 2014). Financial avoidance may be further intensified by weighing the immediate benefits of avoidance (feeling better) more strongly than any delayed outcome of acting (having less problems). In support of this reasoning, research suggests that when experiencing financial scarcity, people are more likely to discount future outcomes (Haushofer & Fehr, 2014; Hilbert et al., 2022b; Ruggeri et al., 2022; Sharma et al., 2023).

In line with this logic, previous longitudinal research spanning more than two years has shown that financial scarcity and financial avoidance have a prospective association with each other (Hilbert et al., 2022a). That is, initial high levels of financial scarcity had a positive association with an increase in financial avoidance more than two years later, and vice versa. Although these findings can help to explain psychological poverty traps (see also Haushofer, 2019), more research is necessary to establish the causal order of the effects. Therefore, we conducted a laboratory experiment in which we investigated whether induced financial scarcity indeed causes financial avoidance.

## Eye tracking and avoidance

In the current study, we induced financial scarcity with the Household Task and examined financial avoidance by measuring gaze patterns with an eye-tracker. The benefit of eye-tracking is that it is an unobtrusive physiological measure, that allows to assess where people look at while they engage (Holmqvist et al., 2011). Thus, unlike more explicit avoidance measures like survey questions, it is less sensitive to experimenter effects and socially desirable responses. Importantly, previous research has shown that gaze patterns are indicative of where people direct their attention to (Carrasco, 2011, Findlay & Gilchrist, 2003; Wedel & Pieters, 2008). More specifically, it has been shown that fixations are indicative of where they focus their attention, and that attentional avoidance is associated with less fixations on the stimulus (see also Borozan et al., 2022).

To date, most eye-tracking research on avoidance has been conducted in patients. For example, research shows that people with anorexia nervosa avoid looking at pictures of food (Giel et al., 2011), people with arachnophobia avoid looking at pictures of spiders (Rinck & Becker, 2006), and people with social anxiety disorder are less likely to look others in the eye (Chen & Clarke, 2017; Weeks et al., 2019). When confronted with a stimulus that depicts the object of their phobia, patients generally show a gaze pattern in line with the hypervigilance-avoidance hypothesis (Pflugshaupt et al., 2005). Following this hypothesis, as compared with non-phobic controls, phobic people fixate quicker on the feared stimulus in the orienting phase after stimulus presentation (hypervigilance), and subsequently, spend less time looking at the feared stimulus (avoidance). This reasoning indicates that after an orienting phase, top-down processes can regulate attention, and therewith gaze-patterns, away from aversive stimuli (see also Lang et al., 1997).

This is supported by findings in non-phobic samples. For example, across multiple studies in a current preprint, budget sizes influenced how people allocated their visual attention (Tomm et al., 2023). The findings suggest that participants with a smaller budget might have looked more at prices but at the same time might have neglect potential discounts that were presented in the visual periphery. In a different study on temporal discounting, it was found that visual attention predicted behavior (Amasino et al., 2019). Participants that preferred sooner and smaller gains (i.e., short-term focus) looked more at temporal information and disregarded information on the amount of the gains, while participants that preferred larger and later gains (i.e., long-term focus) looked more at the amount information and disregarded temporal information. In addition, participants presented with a political advertisement alongside a control stimulus avoided to look at the advertisement if it was inconsistent with their partisan ideology (Schmuck et al., 2020). Likewise, social media users who were not interested in politics spent less time looking at political posts (Bode et al., 2017) and smokers spent less time looking at health warnings on cigarette packages (Maynard et al., 2014). Together, these studies show that eye-tracking can be a valuable tool to measure attentional disengagement from aversive stimuli.

## The present research

In the current study, we used an eye-tracking experiment to test whether financial scarcity leads to financial avoidance. To induce financial scarcity, participants engaged in a task called “Household Task” that simulated the management of a household’s monthly finances. During the task, participants managed the finances of a household over several rounds by earning income and paying expenses. Participants’ financial resources were either insufficient (financial scarcity condition) or sufficient (financial abundance condition) to deal with the financial demands of the situation. Specifically, between conditions, we manipulated financial scarcity by varying whether participants accumulated debts or savings. To assess financial avoidance, participants were presented simultaneously with two letters at the end of each round. One letter constituted the financial stimulus, indicating that an additional expense had to be paid (i.e., expense letter). The other letter was unrelated to participants financial situation and served as a control stimulus.

We chose the following two gaze measures to assess the extent to which participants avoided looking at the expense letter: First, we measured the time it took participants to first fixate on the amount that had to be paid stated on the expense letter. Here, we assumed that as an orienting response after stimulus onset, participants would first read the titles of the two letters. Then, after finding out that one letter was an additional expense, we expected that participants experiencing scarcity would avoid looking at the detailed information stating the amount that had to be paid. This reasoning is in line with prior research on scanpaths of print and online newspapers (Holsanova et al., 2006; Bucher & Schumacher, 2006), websites (Buscher et al., 2009), and printed advertisements (Lohse, 1997), suggesting that people first look at headlines and larger font sizes as an orientation for which content to further direct their attention to (see also, Rahal & Fiedler, 2019). Thus, Hypothesis 1a stated that compared to participants in the abundance condition, participants in the scarcity condition show a longer time to first fixation on the amount stated on the expense letter. Second, we assessed participants’ total fixation duration on the whole expense letter. This was included as an overall measure for attentional disengagement. This measure is commonly used to assess the distribution of attention towards stimuli in research on financial decision-making (Borozan et al., 2022). Thus, Hypothesis 1b stated that compared to participants in the abundance condition, participants in the scarcity condition have a lower relative fixation duration (i.e., proportional dwell time) on the whole expense letter compared to the whole screen.

We also included a behavioral measure of financial avoidance. This entailed that participants could decide to either pay the additional expense directly or to delay its payment. Thus, Hypothesis 2 stated that, as compared to the abundance condition, participants in the scarcity condition are more likely to decide to delay the payment of the additional expense.

## Method

We preregistered our hypotheses, method, and analysis plan on the Open Science Framework (OSF, 10.17605/OSF.IO/SJRWC). All materials, data, and analyses scripts are openly available in the online supplement. The study was approved by the Ethics committee of Leiden University under the number 2019-11-01-M.K.Noordewier-V1-1946.

### Participants and design

We recruited 62 undergraduate students of Leiden University with normal or corrected eyesight as participants for the experiment (*M*_age_ = 23.03 years, *SD*_age_ = 3.09; 54 females, 8 males). We conducted a preregistered sequential analysis with adjusted alpha levels (Lakens, 2014) and decided to stop data collection at this point in favor of collecting the full sample of 100 participants.^1^ To retain a total false positive rate of 5%, the adjusted alpha level for all hypothesis tests was set to α = 0.031 (for calculation of adjusted alpha levels, see online supplementy).

Participants completed the study in individual sessions and were randomly assigned to one of two conditions of a mixed two-factorial design, with Financial Resources (scarcity, abundance) manipulated between participants. In each condition, there were 31 participants. The number of Rounds (one to six) of the Household Task was a within factor. Financial avoidance was measured each round with the time to first fixation on the amount on the expense letter (for Hypothesis 1a) and the total fixation duration on the whole expense letter (for Hypothesis 1b), as well as the decision to pay the extra expense directly or delay the payment (for Hypothesis 2).

### Setup and apparatus

The experiment was programmed in E-prime (version 3.0). Participants completed the study on a laptop with a 15″ wide screen with full HD resolution in the video laboratory of Leiden University. Gaze data was assessed with a Tobii X2-60 eye-tracker. The tracker uses an unobtrusive infrared camera system mounted on the laptop screen, allowing for free head movements. Gaze data was sampled at a rate of 60hz, matching the refresh rate of the laptop screen.

In line with our pre-registered exclusion criteria, we excluded gaze data from the analyses case wise for each round if the valid gaze percentage was lower than 75%. This led to an exclusion of data for 70 rounds, which was 18.8% of the total rounds^2^. The total number of rounds with valid gaze data was 302, clustered in 58 participants.

### Procedure

Participants first gave informed consent, after which they were seated approximately 60 centimeter in front of the laptop screen and the eye-tracker was calibrated. Then, they were introduced to the Household Task, which is a task where participants have to manage the finances of a household. The task can be used to manipulate financial scarcity in a setting of household finances (Hilbert et al., 2022a). Participants completed a practice round to familiarize themselves with the task.

The Household Task consisted of six rounds presented in random order. A round resembled a period of one month in which participants had to earn an income by doing a “monthly work shift”, pay their regular monthly expenses, and respond to mail they received. Each round started with an overview of their expenses. The overview first showed the total amount of expenses, and then listed the expenses for four separate sub-categories (i.e., housing, education, shopping, other). After previewing their expenses, participants continued with an effort task, which represented their work shift. For this effort task, participants were presented with 15 sliders that were distributed across the screen. Sliders ranged from 0 to 100 and displayed the number of the current position of the slider beneath it. Participants were given 30 s to adjust the sliders such that the indicator was set to the middle position (adapted from Gill & Prowse, 2011). They received a fixed income for completing their work shift and a bonus income for each slider they adjusted correctly. Subsequently, they were shown their total income and asked to confirm the payment of their expenses of that round. Then, to measure financial avoidance in the form of attentional disengagement, participants were presented simultaneously with an expense letter and a control letter for 15 s during which we assessed their eye movements. At the end of each round, participants made a binary decision on how to respond to each of the letters, one constituting the behavioral measure of financial avoidance and the other one being a filler task. Then, participants continued with the next round of the Household Task.

After six rounds, the Household Task ended and participants filled in a manipulation check measure and were informed about their earnings and debriefed. Participation took approximately 30 min. Participants were compensated in two ways: First, all participants received incentivized payment based on their final balance in the Household task (up to €3.00). As such, the incentivized payment was dependent on participants performance during the task and their experimental condition (see below). Second and unrelated to their performance or experimental condition, participants received either €4.00 or course credit as a show-up fee.

### Financial scarcity manipulation

To manipulate financial scarcity, we varied participants’ income in the Household Task between conditions. In the scarcity condition, participants had a fixed income of €880 while in the abundance condition, participants had a fixed income of €1204. In addition, participants received a bonus income of €5 for each slider they adjusted correctly^3^. The total income per round thus ranged from €880 to €955 in the scarcity condition and from €1204 to €1279 in the abundance condition. Expenses were based on average expenses for Dutch students and ranged from €1040 to €1120 between rounds, equally across conditions. Each round, participants in the scarcity condition accumulated debts with an average of -€175 (*SD* = 8.27), and participants in the abundance condition accumulated savings with an average e of +€137 (*SD* = 10.5).

### Financial avoidance measures

**Fig. 1:**
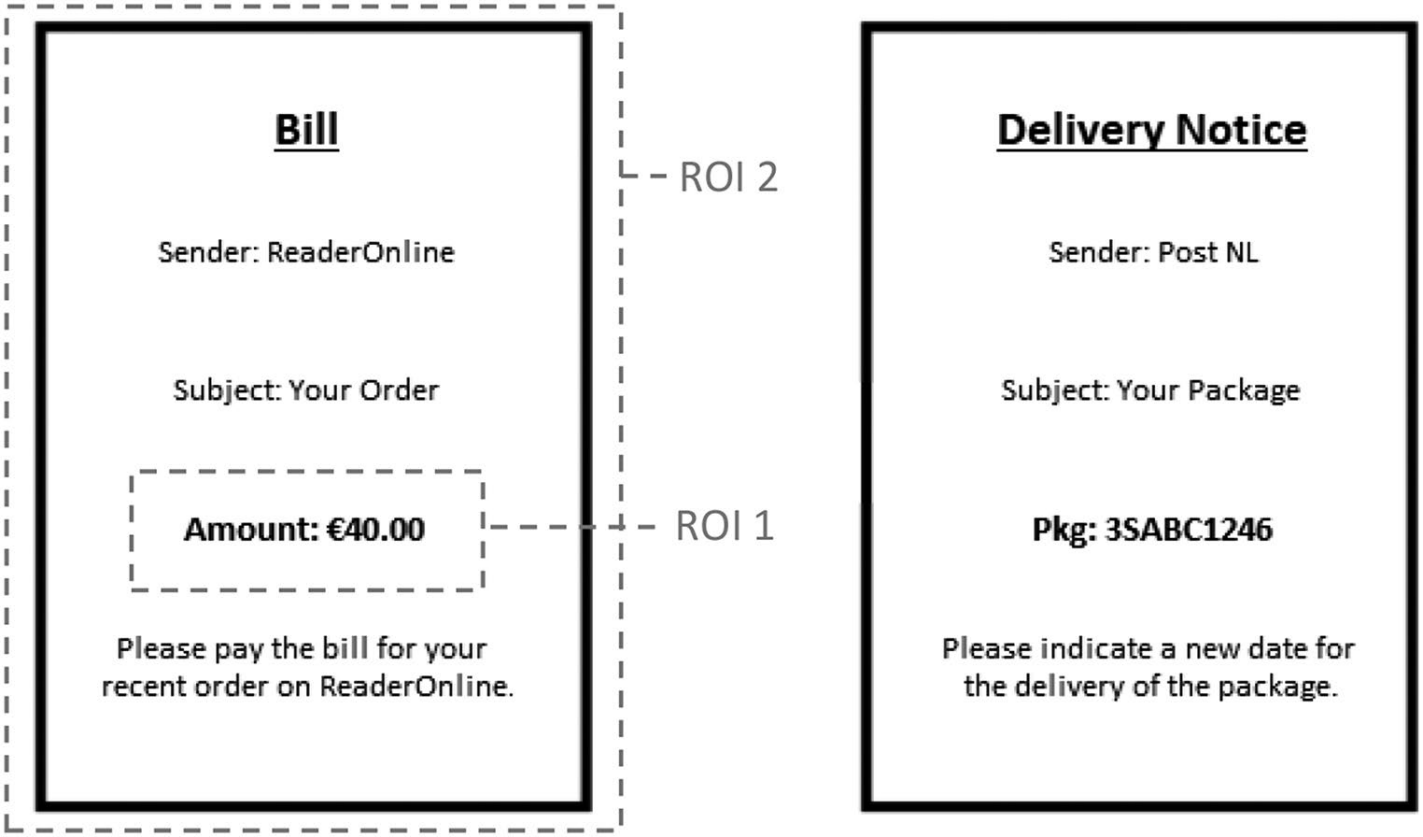
Example stimulus for gaze measurements. The left letter shows an expense, the right letter shows a control stimulus. The two regions of interest (ROI) are highlighted

Each round, after paying expenses and earning income, participants received two letters. One letter indicated that an additional expense had to be paid, the other letter served as a control stimulus (see Fig. 1). The letters were displayed next to each other in randomized position and structured as follows: The header was written in bold, underscored, had a larger font size, and indicated whether the letter informed about a due payment (e.g., bill, invoice) or something non-financial (e.g., delivery notice, registration deadline). The first line stated the sender of the letter and the second line stated the subject of the letter. The third line was in bold and indicated the amount that had to be paid (for the additional expense) or other numerical information (for the control stimulus). Below that, a short sentence asked participants to react to the letter (e.g., please pay the bill).

### Gaze measures

The two letters were displayed simultaneously on screen for 15 s, during which we assessed participants’ gaze data. The position of the letters was randomized (left or right). Two regions of interest (ROIs) were defined to code the fixations of participants (Fig. 1). The first ROI was the amount of money that had to be paid stated on the expense letter. As a measure for Hypothesis 1a, we calculated the time to first fixation on this ROI. To count as fixation on the ROI, the fixation duration had to be at least 100 milliseconds. The second ROI was the whole expense letter. As a measure for Hypothesis 1b, we assessed participants ‘proportional dwell time’ on this ROI by calculating the relative total fixation percentage on the expense letter compared to all valid fixations on the screen (Maynard et al., 2014; Schmuck et al., 2020).

### Behavioral measure

Each round, after being presented with the two letters, participants made a binary decision about each of them (for a full list of stimuli, see online supplement). The decisions were made on individual screens in randomized order. Regarding the expense letter, participants were asked whether they wanted to pay the expense (e.g., “Would you like to pay the bill of €39.99 from city hall now or later?”) and could choose to either “Pay now” or to “Pay later”. If participants decided to pay the expense directly, the respective amount was deducted from their balance. If participants decided to pay the expense later, they were presented with it again at the end of the experiment. Delaying payment was not associated with a risk or cost. The total count of delayed payments constituted the dependent variable for Hypothesis 2 and could range from 0 (each round paid directly) to 6 (each round paid later).

The decisions about the control letter were included merely to avoid the experimental design steering the attentional focus on the expense letters over rounds. Participants were asked a question about the control letter (e.g., “Please choose a course for which you want to register) and made a binary decision (e.g., “Inferential Statistics” or “Clinical Psychology”). These decisions were inconsequential for the outcome of the experiment and therefore not analyzed. At the end of each round, participants were shown their updated balance.

### Manipulation check

To test whether the difference in balance between conditions successfully manipulated participants’ experience of financial scarcity, we included a four-item self-report measure of financial scarcity at the end of the experiment. Participants reported their experience of financial scarcity throughout the task, with two positively coded items (“I worried about my financial situation”, “I felt stressed about my financial situation”) and two negatively coded items (“I felt I had enough money”, “I felt I had control over my finances”). The items were adapted from Hilbert et al. (2022a) and assessed on a seven-point Likert-scale, ranging from 1 = *totally disagree* to 7 = *totally agree*. The measure showed high internal consistency (Cronbach’s α = 0.87).

## Results

### Manipulation check

Confirming that the manipulation was successful, participants in the scarcity condition reported a stronger experience of financial scarcity (*n* = 31, *M* = 5.98, *SD* = 0.89) compared to participants in the abundance condition (*n* = 31, *M* = 2.82, *SD* = 1.07, *t*[57.9] = 12.63, *p* < .001, *g* = 3.21).

As a robustness check, we also tested whether the manipulation affected participants motivation to perform well during the task, which might threaten the validity of our findings. Importantly, there was no evidence suggesting that the experimental condition affected participants performance in the effort task, as there was no significant difference between correctly adjusted sliders in the scarcity condition (*M* = 53.1%, *SD* = 6.71%) and the abundance condition (*M* = 51.9%, *SD* = 9.50%), *t*(54.0) = 0.55, *p* = .585, *g* = 0.14. This indicates that although incentives differed between conditions, participants were equally motivated to perform during the task.

### Gaze data

#### Time to first fixation

We predicted that financial scarcity might induce a tendency to avoid learning negative financial information. Hypothesis 1a stated that when presented with the two letters on the screen, participants in the scarcity condition might delay looking at the amount on the expense letter that stated how much was needed to be paid (ROI 1, see Fig. 1). To test this hypothesis, we fitted a linear mixed model (REML) with time to first fixation (in seconds) on the amount of the additional expense as dependent variable. In line with our preregistered analysis plan, we included random intercepts for participants but no random slopes in the model, as the latter would have decreased model fit. In addition, the model included a fixed level-1 factor for the number of rounds, a fixed level-2 factor for the experimental condition, and a cross-level interaction between the two fixed effects for exploratory purposes. Overall, the total variance explained was *r*²_conditional_ = 0.06. The intra-class-correlation (ICC) was low, *r* = .06. The variance explained by the fixed effects was *r*²_marginal_ = 0.00. The fixed effects are displayed in Table 1.

**Table 1:** Fixed effects for linear mixed model predicting time to first fixation on the amount of the expense letter

			95% CI				
Effect	Estimate	*SE*	Lower	Upper	df	*t*	*p*
(Intercept)	4.19	0.23	3.74	4.64	45.3	18.20	< 0.001
Condition	-0.17	0.46	-1.07	0.73	45.3	-0.37	0.715
Round	0.04	0.12	-0.19	0.27	229.1	0.32	0.750
Condition ✻ Round	0.15	0.23	-0.31	0.60	229.1	0.63	0.530

Not supporting Hypothesis 1a, the time to first fixation on the amount of the additional expense was not significantly higher in the scarcity condition compared to the abundance condition, *b* = -0.17, *p* = .715. Also not significant were the fixed effect of Round, *b* = 0.04, *p* = .750, and the cross-level interaction, *b* = 0.15, *p* = .530.

#### Fixation duration

We predicted that financial scarcity might lead to attentional disengagement from negative financial information. Hypothesis 1b stated that when presented with the two letters on screen, participants in the scarcity condition might spend less time looking at the whole expense letter (ROI 2, see Fig. 1) relative to the rest of the screen. To test this hypothesis, we fitted the same linear model with relative dwell time on the whole expense letter as dependent variable. Overall, the total variance explained in the final model was *r*²_conditional_ = 0.31. The intra-class-correlation (ICC) was moderate, *r* = .30. The variance explained by the fixed effects was *r*² = 0.01. The fixed effects are displayed in Table 2.

**Table 2:** Fixed effects for linear mixed model predicting relative fixation duration on the expense letter

			95% CI				
Effect	Estimate	*SE*	Lower	Upper	df	*t*	*p*
(Intercept)	29.98	0.87	28.28	31.69	53.8	34.40	< 0.001
Condition	1.54	1.74	-1.88	4.95	53.8	0.88	0.382
Round	-0.43	0.28	-0.98	0.13	248.5	-1.50	0.135
Condition ✻ Round	-0.68	0.57	-1.78	0.43	248.5	-1.19	0.234

Not supporting Hypothesis 1b, the fixation duration percentage on the expense letter was not significantly lower in the scarcity condition compared to the abundance condition, *b* = 1.54, *p* = .382. Also not significant were the fixed effect of Round, *b* = -0.43, *p* = .135, and the interaction of the cross-level interaction, *b* = -0.68, *p* = .234.

Taken together, the gaze data provided no evidence suggesting that the experience of financial scarcity increased attentional disengagement from negative financial information.

#### Behavioral data

Next, to test Hypothesis 2, we fitted a logistic mixed model with a binomial dependent variable, indicating for each round whether participants decided to pay their additional expense directly or delayed their payment. The model contained random intercepts for participants, random slopes for participants across Rounds, and the same fixed effects as the previous models. Overall, the total variance explained was *r*²_conditional_ = 0.67. The intra class correlation (ICC) was moderate, *r* = .38. The variance explained by the fixed effects was *r*²_marginal_ = 0.35. The fixed effects are displayed in Table 3.

**Table 3:** Fixed effects for generalized mixed model (log) predicting decisions to pay expense directly or later

				95% exp(B) CI			
Effect	Estimate	*SE*	exp(B)	Lower	Upper	*z*	*p*
(Intercept)	-2.00	0.35	0.14	0.07	0.27	-5.72	< 0.001
Condition	3.71	0.68	41.00	10.84	155.13	5.47	< 0.001
Rounds	0.12	0.17	1.13	0.81	1.58	0.71	0.478
Condition ✻ Rounds	-0.11	0.33	0.90	0.47	1.72	-0.33	0.741

Confirming Hypothesis 2, as compared to participants in the abundance condition, those in the scarcity condition were more likely to delay payment of their additional expense, γ = 3.71, *p* < .001. This was equivalent to a total *M* = 2.84 (*Median* = 3, *SD* = 1.70) of delayed payments in the scarcity condition and total *M* = 0.42 (*Median* = 0, *SD* = 0.85) of delayed payments in the abundance condition. The fixed effect of Rounds was not significant, γ = 0.12, *p* = .478. Likewise, the interaction between the two fixed factors was not significant, γ = -0.11, *p* = .741.

Thus, the behavioral data supported the hypothesis that financial scarcity increases the preference to delay dealing with one’s finances.

## Discussion

Previous longitudinal research showed that when experiencing financial problems, people are more likely to avoid potentially negative financial information and delay making financial decisions (Hilbert et al., 2022a; see also Gilbert et al., 2022). Here, we experimentally investigated whether financial scarcity increases financial avoidance. That is, depending on experimental condition, participants either accumulated household debts or savings, which served as a manipulation of financial scarcity and financial abundance, respectively. Then, participants received an additional expense letter in their mail. We tested whether participants who experienced financial scarcity would attentionally disengage from the expense letter and delay their payment. We measured attentional disengagement with an eye-tracker in two ways: First, we assessed the time it took people to look at amount they had to pay stated on the expense letter. Second, we assessed the proportional time people spent looking at the expense letter while it was presented next to a control stimulus. Then, as a behavioral measure of financial avoidance, we gave participants the option to delay the payment of the bill without additional cost until the end of the experiment. The eye-tracking data did not support our hypothesis that financial scarcity leads to attentional disengagement. The behavioral data, however, supported our hypothesis that financial scarcity increases the likelihood to delay the payment of bills.

Several methodological and theoretical considerations offer insights into the findings regarding the hypothesized effect of financial scarcity on attentional disengagement. Notably, while exploring the data, it’s evident that our eye-tracking measurements exhibited varying degrees of precision. Data for the first hypothesis (H1a) concerning the time taken for participants to first fixate on the expense letter may have been influenced by the relatively small size of the region of interest (ROI). This could potentially lead to misclassifications of fixations, a challenge acknowledged in prior research (Holmqvist et al., 2022). Consequently, it is possible that null effect in gaze data can be attributed to random error, potentially impacting the outcome for H1a. Conversely, the data for the second hypothesis (H1b) relied on the percentage of all fixation durations within a significantly larger ROI. This stability is reflected in the substantial portion of explained variance in the gaze data by the predictors and a notable interclass correlation coefficient (ICC) (Bliese, 2000; James, 1982). Therefore, it’s reasonable to infer that the robustness of the results for H1b is less susceptible to the influence of noisy data.

It is thus more plausible that under the current circumstances, financial scarcity indeed does not lead to attentional disengagement from one’s financial problems. Participants had the option to delay paying their additional expense at no cost and could thereby effectively deal with the problem at hand. This might have provided them with a sense of control over their problematic financial situation and led to the perception that the additional expenses were manageable. Crucially, previous research has shown that a threat leads to disengagement when threat-management resources are lacking (Howell et al., 2014) but not when the threat is perceived to be manageable (i.e., under control; Garrett et al., 2018). That is, the general tendency to avoid negative information (Sweeny et al., 2010) can be attenuated under controllable threat, as all information might be crucial to survival and therefore processed equally. Therefore, we think that the perception of (not) having control over one’s finances might be a crucial moderator that influences whether one’s financial problems are seen as an uncontrollable threat that should be avoided or a challenge that can be overcome. Future research could systematically investigate the potential moderating role of perceived lack of control for the effect of financial scarcity on financial avoidance, for example by introducing and varying an additional cost for the option to delay one’s additional expenses.

The behavioral findings contribute to the literature by establishing a causal relationship between financial scarcity and financial avoidance. These findings extend previous correlational research showing that the experience of financial scarcity is associated with an increase in financial avoidance over time (Hilbert et al., 2022a) and that low economic status is associated with a general avoidance motivation (Gilbert et al., 2022). Here, we showed experimentally that financial scarcity increases financial avoidance behavior. At the same time, we found no evidence suggesting financial scarcity decreases attention to negative, but important, financial information. These findings have relevant implications for policy and practice. When having trouble to make ends meet, people are more likely to delay paying their bills. Crucially, we found this behavioral tendency to be a function of the financial situation, indicating that delayed payments of the indebted or poor can be caused by the circumstance of their financial situation alone. Moreover, we did not find evidence suggesting that this behavioral pattern was explained by attentional disengagement. The effectiveness of interventions might thus be increased when they are aimed at improving the financial circumstances of the financially deprived, in addition to or instead of aiming to “improve” decisions of individuals with financial problems through self-control trainings or financial literacy courses.

Importantly, financial avoidance like we observed in the current study is not necessarily irrational. When needed resources are lacking, it can be sensible to delay payments, especially when the associated costs are low. This contention is in line with recent findings suggesting that people lacking needed financial resources show increased temporal discounting only when it would be rational (Hilbert et al., 2022b; Sharma et al., 2023) and that poor people make equally biased decisions as the rich (Ruggeri et al., 2022, 2023).

## Conclusion

When people have too little financial resources to meet demands, they can experience financial scarcity. Here, we tested whether the experience of financial scarcity during the Household Task would lead to financial avoidance, measured as attentional disengagement from expense letters with an eye-tracker and behavioral avoidance by delaying the payment of expenses. The experiment did not provide evidence in support of the hypothesis that financial scarcity affects how people distribute their attention, as there was no effect of participants’ financial situation on their gaze pattern. However, financial scarcity increased the likelihood to delay paying one’s expenses. This finding extends previous correlational findings by establishing a causal order on the association between scarcity and avoidance.

## Data Availability

All data and analysis code are openly available on the Open Science Framework https://osf.io/zr49x/.

## Footnotes

1 We had to stop recruitment of participants in March 2020 when campus was closed due to the outbreak of the COVID-19 pandemic. Before deciding whether to commence data collection or not, and without prior knowledge of the already collected data, we added the sequential analysis to our pre-registration. Then, we analyzed the data with adjusted alpha levels and decided not to pick up data collection again when students returned to campus in summer 2021.

2 Gaze data quality did not differ between experimental conditions, *t*(360) = -0.09, *p* = .925. Inclusion of these data points did not change the results in a meaningful way (see open materials).

3 Participants were instructed that they would receive both a fixed income and a bonus income, but they were only shown their total income for each round.
